# A Fluorescent Sensor-Assisted Paper-Based Competitive Lateral Flow Immunoassay for the Rapid and Sensitive Detection of Ampicillin in Hospital Wastewater

**DOI:** 10.3390/mi11040431

**Published:** 2020-04-20

**Authors:** Honggui Lin, Feixiang Fang, Jiahui Zang, Jianlong Su, Qingyuan Tian, Ranjith Kumar Kankala, Xuexia Lin

**Affiliations:** 1 Fujian Province Key Laboratory of Ship and Ocean Engineering, Marine Engineering College, Jimei University, Xiamen 361021, China; linhongui36@163.com (H.L.); tianqingyuan@jmu.edu.cn (Q.T.); 2 Department of Chemical Engineering& Pharmaceutical Engineering, College of Chemical Engineering, Huaqiao University, Xiamen 361021, China; fangfeixiangHQU@163.com (F.F.); zjh19980729@163.com (J.Z.); sujianlong_123@163.com (J.S.); ranjithkankala@hqu.edu.cn (R.K.K.); 3 Marine Engineering College, Dalian Maritime University, Dalian 116026, China

**Keywords:** CF-LFI, oligonucleotide probe, ampicillin, hospital wastewater

## Abstract

In this study, a convenient assay method has been developed based on labeled functional nucleic acids (H-DNA) and a competitive fluorescent lateral flow immunoassay (CF-LFI) for ampicillin (AMP) detection. Herein, we designed the tunable AMP probes for AMP detection based on the AMP aptamer, and the secondary DNA fragment. The probes can generate tunable signals on the test line (T line) and control line (C line) according to the concentration of AMP. The accuracy of detection was improved by optimizing the tunable AMP probes. Under the optimal conditions, the linear concentration of AMP detection is ranged from 10 to 200 ng/L with a limit of quantitation (LOQ) value of 2.71 ng/L, and the recovery is higher than 80.5 %. Moreover, the developed method shows the potential application for AMP detection in the hospital wastewater.

## 1. Introduction

Ampicillin (AMP), a beta-lactam antibiotic, has been widely used in the medical, aquaculture, and agriculture-based industries to prevent and treat bacterial infections of *Haemophilus influenzae*, *Escherichia coli*, and *Salmonella* as well as *Shigella* species [1,2]. High concentrations of AMP residues in the contaminated waters often lead to severe health complications of allergic reactions, dyspepsia, and epilepsy [3], requiring effective assay methods for its rapid and sensitive detection. Several on-site tests have been recognized as promising approaches for antibiotic detection owing to specific advantages of low cost and convenience. These antibiotic assays conducted by chemiluminescence [4], fluorescence (FL), electrochemistry, and chromatography exhibited numerous benefits in diverse applications [5,6]. However, they suffer from several disadvantages such as being time-consuming, relying on highly expensive instruments, and insufficient detection of AMP. To meet the demand of rapid detection and high sensitivity, LFI has garnered increasing interest in recent times for the substantial detection of antibiotic residues in water. Chen and his coworkers developed the near-infrared (NIR) FL-based LFI for simultaneous detection of four antibiotic residues and then improved the sensitivity [7]. Although the LFI has demonstrated some superiority on substantial detection, the sensitivity and selectivity of LFI need to be improved to detect AMP due to the following reasons [8,9,10]. First, slight amounts of AMP residues and their degraded products exist in the medical waste samples [11]. Second, varieties of antibiotics such as chloramphenicol (CTC), oxytetracycline (OTC), and tetracycline (TC) are present, resulting in the complex matrix. Third, AMP in water environments also can be adsorbed, hydrolyzed, photolyzed, biodegraded, and so on, which increase the difficulty of qualitative analysis [12]. Finally, AMP is a small molecule, it is more challenging to obtain its high selectivity of antibody comparied to the large molecules such as proteins [3]. Thus, it is of significance to develop a highly-sensitive, selective, simple, and fast detection for AMP assay in the wastewater.

Aptamers are considered as substitutes for the antibody, which have been widely used in biosensors because of their high stability, affinity and specificity, and ease of synthesis [13,14]. Several efforts have been dedicated to the advancements for the development and application of aptamers towards identifying target antibiotics. In our previous reports, the luminescent carbon nanoparticles based aptasensors were fabricated for the detection of kanamycin (KA) and oxytetracycline (OTC) residues [15,16]. In another case, Rozlosnik and colleagues successfully analyzed AMP and KA using aptamer-assisted electrochemical microfluidic technology [17]. Furthermore, by applying LFI and aptamer, Deigner et al. developed an AMP detection method through aptamer-C-reactive protein cross-recognition [3]. Despite the critically high sensitivity for the antibiotic detection [18], the data from aptamer-based LFI techniques often suffer from problems such as the background interference, the fluctuation of detection conditions, and the inadequate selectivity [19,20]. Rather than changing the physical parts, CF-LFI can be used to reduce the interference of the sample matrix, reduce the deviation, and improve quantitation ability of LFI due to the tunable AMP probes [21,22,23]. A unique change is made in the conventional design of competitive LFA by introducing the tunable AMP probes, and leading to producing the self-calibration signals. This self-calibration method is based on a ratiometric approach for detection of AMP, which combines the theory of principle of immune competition. The optical signals were obtained according to the signals of T line and C line. Then, the total optical signals including the test signal and control signal carried on taking the internal parameters as the initial value. After that, the accurate optical signals ratios (FL_T/C_) were calculated by the internal parameters, test signal, and control signal. Therefore, this ratiometric strategy can be self-calibrated. The ratiometric approach could efficiently eliminate not only the background interference, but also the fluctuation of detection conditions arising from operation experiment or instrumental factors, which can greatly improve the reliability of AMP detection in real samples. 

In this work, we reported a simple strategy for high-sensitive assay of AMP in the hospital wastewater depending on CF-LFI and tunable aptamer probes. As a proof-of-concept, the tunable probe (H-DNA) was fabricated with AMP aptamer, the conjugating DNA fragment, and a secondary DNA fragment. These tunable probes enabled bonding test DNA (T-DNA) and control DNA (C-DNA), resulting in the FL intensity changes at T line and C line. Notably, a secondary DNA fragment in the H-DNA was designed not only for the competitive LFI but also for a reference object to reduce the external factor as well as matrix interferences. Finally, the designed assay was applied to detect AMP in the hospital wastewater and validated in terms of the selectivity, linearity, sensitivity, and recovery. 

## 2. Materials and Methods 

### 2.1. Materials

Ampicillin trihydrate, bovine serum albumin (BSA), sodium alginate, agarose (low gelling temperature) and triton X-100 (polyethylene glycol octyl phenyl ether) were obtained from Sigma Co., Ltd. (St. Louis, MO, USA). Electrophoresis gel stain Gold View I type nucleic acid stain, DNA loading buffer, marker I, and DNA ladder were purchased from Beijing Solarbio Science & Technology Co., Ltd. (Beijing, China). Tris (hydroxymethyl) methyl aminomethane, hydrochloric acid, sodium citrate, sodium chloride, sodium hydroxide, potassium chloride, potassium phosphate monobasic, and dibasic sodium phosphate were purchased from Sinopharm Group Chemical Reagent Co., Ltd. (Shanghai, China). The nitrocellulose (NC) membrane attached to a backing card, absorbent pad and glass fibers were purchased from Jiening Biological Technology Co., Ltd. (Shanghai, China). The ultrapure water (18 MΩ·cm^−1^) was obtained from a Milli-Q water purification system (Millipore Corporation, MA, USA). All chemicals used in this study were of analytical reagent grade. In this study, the AMP aptamer was prepared according to previously reported research [23], and the DNA samples used in this study were purchased from Sangon Biological Engineering Technology & Services Co., Ltd. (Shanghai, China). The AMP FNAs have the following sequences:

Test DNA (T-DNA): 5′-CCG CTA TAC AAC CGC CCG-C6-bio 3′.
Control DNA 1 (C-DNA 1): 5′-GTC AGA TGA ATT CGT GTG AGA AAA A-bio-3′.
Control DNA 2 (C-DNA 2):5′-CCC ATC CCG CCC AAC CCA AAA A- bio3′.
HEX label DNA 1 (H-DNA 1): 5′-*GCG GGC GGT TGT ATA GCG G*TT TTTTCT CAC ACG AAT TCA TCT GAC-HEX-3′.
HEX label DNA 1’ (H-DNA 1’): 5′ -HEX -*GCG GGC GGT TGT ATA GCG G*TT TTTTCT CAC ACG AAT TCA TCT GAC -3′.
HEX label DNA 2 (H-DNA 2): 5′-*GCG GGC GGT TGT ATA GCG G*TT TTT TGG GTT GGG CGG GAT GGG-HEX-3′.

T-DNA is an AMP aptamer complementary chain. The italicized bold letters of H-DNA represent an AMP aptamer sequence. The underlined letters of H-DNA represent the secondary DNA fragment that is the recognition sequence of C-DNA. Hexachloro-6-carboxyfluorescein (HEX) was used as a fluorescent dye due to its low background signal in the NC membrane.

The buffers listed below were used in this study. The coating buffer was 10 mmoL Tris-HCl buffer (pH 7.4) containing a streptomycin-biotinylated DNA probe. The streptomycin-biotinylated DNA capture was prepared as follows: 2 mg/mL streptomycin was mixed with 10 μg/mL biotinylated DNA capture probe for 1 h at 37 °C. Then, the mixture solution was purified by ultrafiltration centrifugation at 6000 rpm with a centrifugal filter (cut off 30,000, Millipore) for 30 min. The purified solution was dissolved in 10 mmoL Tris-HCl buffer (pH 7.4) for further experiments.

The blocking buffer was prepared by adding 1.5% BSA (W/V), 0.06% sodium alginate (W/V) and 1% Triton X-100 (V/V) to 0.01 M phosphate buffer (pH 7.4). The washing buffer was prepared by adding 1% Triton X-100 to 10 mmoL of PBS (pH 7.4). To obtain the fluorescent intensity of the test strip, the images were captured by a fluorescence and chemiluminescence imaging system (Qin Xiang Instrument Co., Ltd., Shanghai, China). 

### 2.2. Preparation of CF-LFI

**Figure 1:**
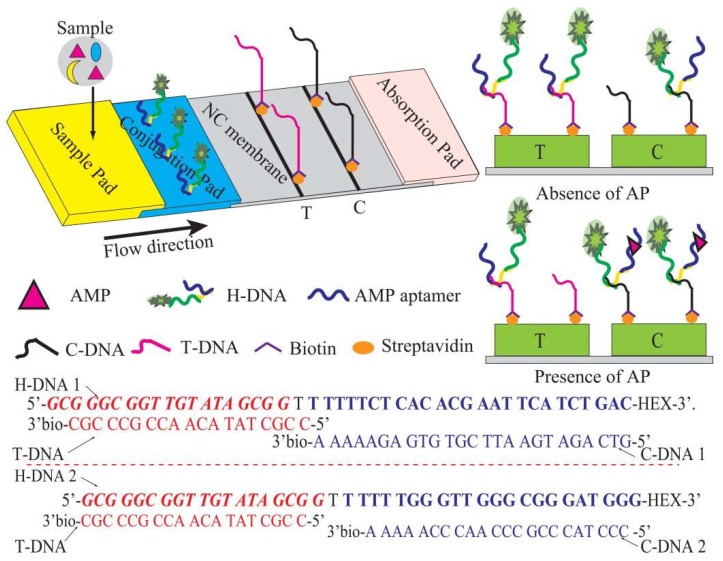
Schematic of CF-LFI for AMP detection.

The structure of the test strip is as shown in Figure 1. Conjugate, as well as sample pads, were prepared using glass fiber. Prior to the treatment, these pads were treated with the blocking buffer overnight and dried at 37 °C for 15 min. A certain concentration of H-DNA solution was dispensed onto each conjugate pad (0.3 cm × 0.5 cm) by an SP-20E automatic sampling instrument (Kezhe Biochemical Technology Co., Ltd., Shanghai, China). The streptomycin-biotinylated T-DNA and C-DNA in the coating buffer were separately coated on the NC membrane as the T line and C line by using the SP-20E automatic sampling instrument. After drying at 37 °C for 2 h, the sample pad, conjugate pad, NC membrane, and absorbent pad were pasted on the PVC board to be assembled into the test strip. The arrangement of the components was such that each part was overlapped 2 mm to ensure the migration of the solution. The assembled pads were arranged in pieces (0.3 × 6.0 cm^2^), installed in the plastic shell, and stored in a desiccator at room temperature.

### 2.3. Sample Preparation

Hospital wastewater contains many impurities such as particles, proteins, metal ions, and nucleases. Particles can block micropores in test strips, resulting in inadequate diffusion. Sample pretreatment is applied to reduce the protein and nucleases interference from hospital wastewater. The sample pretreatment method can be further used in the analysis of other samples which are from the different environment or different kind of sample such as milk. In detail, the sample (5 mL) hospital wastewater was centrifuged at 4000 rpm for 10 min and the supernatant was collected. The supernatant was added to 500 μL of trichloroacetic acid (vortexed for 1 min). Then, it was placed on a vortex shaker for 1 min. After that, the mixture solution was centrifuged at 6500 rpm for 10 min. The supernatant was collected and filtered through a 0.22 μm microporous membrane. The filtrate was adjusted to pH 7.4 with NaOH solution (1 M) and stored at −20 °C for further experiments. In this work, the addition of trichloroacetic acid to the sample can make the nuclease and other protein aggregate and precipitate. After the filtration, their interference can be greatly reduced.

### 2.4. Sample Analysis

AMP analysis was performed by inserting the developed strip into AMP standard solution or the pretreated sample solutions. With the aid of the absorbent pad, the solution could move along the NC membrane to react with H-DNA in the conjugate pad. In the case of the absence of AMP in the sample solution, most of H-DNA would reach the T line and could be captured by the immobilized T-DNA, resulting in the highest fluorescent signal. In another case of its presence in the sample solution, the H-DNA could react with the available AMP, resulting in reduced fluorescent signal in T line and enhanced fluorescent signals in the C line because the H-DNA-AMP conjugation could be captured by C-DNA but not T-DNA. After adding sample for 5 min, the assembled pads were detected at the characteristic excitation wavelength (538 nm) using a fluorescence and chemiluminescence imaging system. The optical signal was relevant to gray analysis intensities and can be calculated using the gel analysis of Image J Soft [24,25]. 

### 2.5. Data Analysis

The assay solution and samples were run in CF-LFI strip, and the FL intensities were measured. Calibration curves were obtained by plotting Y against the AMP concentration and fitted to the linear equation of Y-X as follows:

$$Y=\frac{BV}{A-BV}C_{X}=\frac{BV}{B_{0}V}C_{X}=\frac{B}{B_{0}}C_{X},$$

where *B*_0_ is the FL intensity at C line, *B* is FL intensity at T line, *A* is the total FL signal value, *V* is the area of detection line, and *C_X_* is the AMP concentration. Notably, the areas of T line and C line are the same.

### 2.6. Method Validation

The parameters of selectivity, linearity, sensitivity, and recovery of the method were evaluated. The recovery was analyzed by respectively spiking AMP standard solution to hospital wastewater samples. The working standard solutions at the concentrations of 1, 10, 20, 50, 100, 150, 200 and 300 ng/L were obtained by diluting the stocking solution with ultrapure water. LOQ was determined by calculating the minimum amount of AMP that could be markedly distinguished from the blank sample signal (S_0_) (mean binding at S_0_ − (10 × SD), 3 replicates). SD means standard deviation. The recoveries and relative standard deviations (RSDs) were tested by spiking 20 and 150 ng/L AMP standard solution into the hospital wastewater samples. In order to study the matrix effect, the real sample was diluted 5-fold and 10-fold with the ultrapure water and then the recoveries were analyzed.

## 3. Results and Discussion

Working principle of CF-LFI. 

To develop a sensitive and reliable CF-LFI method for AMP analysis, different H-DNA probes were designed, and the paper-based CF-LFI system was fabricated. At the outset, the FL signal (FL_T/C_) was calculated by the amounts of fluorescent intensities at the T line and C line. In this study, a certain amount of H-DNA was immobilized on the conjugate pad to capture AMP (Figure 1). In the absence of AMP, the H-DNA is captured by T-DNA, resulting in high fluorescence signal in T line and weak fluorescence signal in C line. In the presence of AMP, the H-DNA-AMP complexes are captured by the C-DNA complex and excess H-DNA is captured by T-DNA, resulting in high fluorescence signal in C line and weak fluorescence signal in T line. Hence, the FL_T/C_ value can be obtained. In addition, the secondary DNA fragment in the H-DNA is also designed as a reference. Thus, the deviation for every analysis and the matrix interference can be significantly reduced due to the application of H-DNA and FL_T/C_ for LFI analysis.

### 3.1. Designed AMP Probes

**Figure 2:**
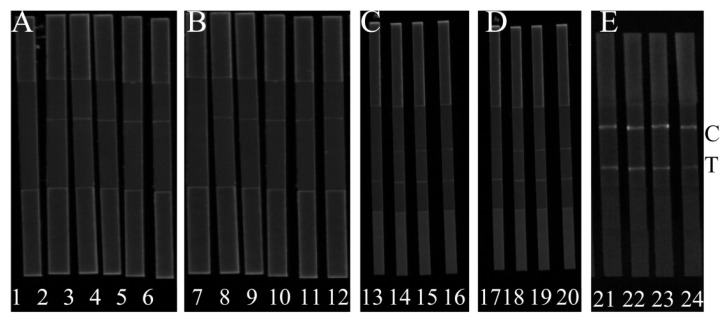
Evaluation of probes (H-DNA, T-DNA, C-DNA) for AMP detection.

For decreasing the deviation, and confirming the effectiveness of the developed method, two different kinds of the secondary DNA fragment were designed and the positions of fluorescent dye HEX were also optimized. Figure 2A shows that H-DNA 1 could interact with T-DNA, AMP and C-DNA 1 and H-DNA 1-AMP complex can interact with C-DNA 1. In the presence of AMP, the FL intensities at the C line were strongly enhanced based on the usage of H-DNA 1, while the FL intensities were greatly decreased at the T line in Figure 2C. In the absence of AMP, the FL intensities at the C line were weak and strongly enhanced at T line. Moreover, the FL at the T line was gradually reduced by increasing the concentration of AMP. Contrarily, the FL at the C line was gradually enhanced. Together, the FL_T/C_ was gradually declined with the increase of AMP. Although the change of FL_T/C_ could be observed when H-DNA 2 reacted with C-DNA 2 (Figure 2B), the changes of FL_T/C_ based on test signal and control signal were slight (Figure 2D). Therefore, H-DNA 1 was used for further experiments. In order to evaluate the high effectiveness of AMP detection, the position of the fluorescent dye HEX in the H-DNA 1 was optimized. The position of the HEX fluorescent dye was used to control luminous signal and can influence the reaction efficiency of H-DNA and AMP. Figure 2E shows that with the application of H-DNA 1’, both the test signal and control signal are higher, and the slight FL_T/C_ change can be observed with the different concentration of AMP, while the obvious change in FL_T/C_ can be observed when H-DNA 1 is applied. We deduced that if HEX is close to AMP then the aptamer could prevent the combination of H-DNA 1’ and AMP, resulting in the low signal change of the developed method. Therefore, H-DNA 1 and C-DNA 1 was used for the following experiments.

Strips 1, 2, 3, 4, 5, and 6 are used to illustrate the feasibility of probes T-DNA, C-DNA 1, and H-DNA 1. Strips 7, 8, 9, 10, 11, and 12 are used to illustrate the feasibility of the probes T-DNA, C-DNA 2 and H-DNA 2. Strips 13, 14, 15, and 16 are coated with C-DNA 1 to analyze AMP with 300 ng/L, 200 ng/L, 100 ng/L, and 50 ng/L, respectively. 

Strips 17, 18, 19, and 20 are coated with C-DNA 2 to analyze AMP with 300 ng/L, 200 ng/L, 100 ng/L, and 50 ng/L, respectively. Strips 21 and 22 is the application of H-DNA 1’ to analyze AMP with 150 ng/L and 200 ng/L, respectively. Strips 23 and 24 is the application of H-DNA 1 to analyze AMP with 150 ng/L and 200 ng/L, respectively. More details are in the supporting information. 

### 3.2. Optimization of T-DNA and C-DNA Concentrations 

**Figure 3:**
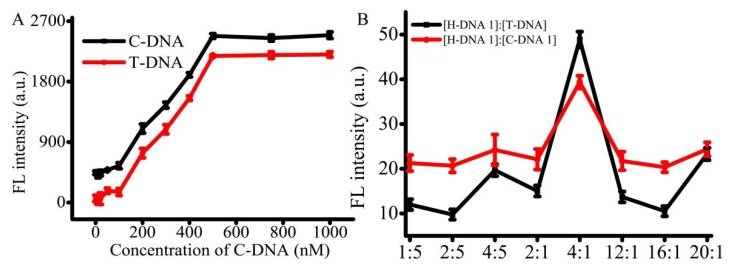
Optimization of DNA conditions. (**A**) FL intensity against various concentrations of C-DNA and T-DNA, and (**B**) various ratios of H-DNA 1 and T-DNA or C-DNA 1.

For the AMP detection, the T-DNA and C-DNA 1 were pre-immobilized on the T line and C line. The use of suitable concentrations of the immobilized T-DNA and C-DNA 1 can improve the efficiency of AMP detection. To address this issue, the immobilized concentrations of T-DNA, C-DNA 1, and H-DNA 1 were optimized. According to government standard guidelines (ref. NT/Y 829-2004), the limit of detection is 1 μg/kg and the linearity range changes from 1 to 80 μg/kg with the dissociation equilibrium around 13.4 nM. The concentration of AMP work solution was set at 1 μg/mL. The concentrations of T-DNA and C-DNA 1 were optimized with H-DNA 1. When the concentration of T-DNA ranged from 0 to 100 nM, the signal was slightly enhanced (Figure 3A). When the concentration of T-DNA ranged from 100 to 500 nM, a good linear relationship was detected with 200 ng/L AMP. Furthermore, the platform appeared when the concentration of T-DNA was in the range of 500 to 1 μM. The results indicated that when the concentration of T-DNA was above 500 nM, T-DNA was superfluous to react with 200 nM of AMP. The trend of C-DNA 1 was similar to that of T-DNA. Therefore, the concentration of both T-DNA and C-DNA 1 were selected as 500 nM, respectively. In addition, in order to get high effective analysis, the concentration of H-DNA 1 was also optimized. Altered ratios of H-DNA 1 and T-DNA or C-DNA were assessed, such as 1:5, 2:5, 4:5, 2:1, 4:1, 12:1, 16:1, and 20:1. The optimum concentration ratio of H-DNA 1 to T-DNA was 4:1 (Figure 3B), indicating the optimum concentration of H-DNA 1 is 2.0 μM. The trend of C-DNA 1 is similar to that of T-DNA. Thus, the concentrations of T-DNA, C-DNA 1, and H-DNA 1 were set at 500 nM, 500 nM, and 2.0 μM, respectively. 

### 3.3. Effect of Detection Time 

**Figure 4:**
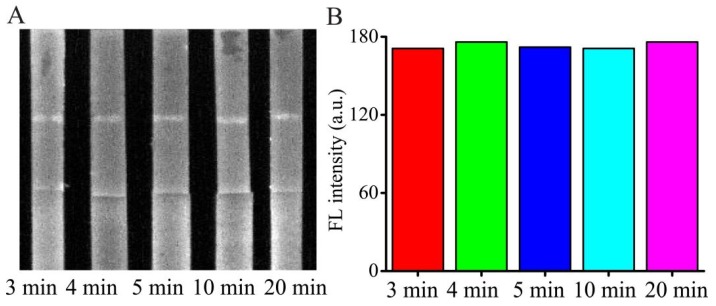
Optimization of the detection time. (**A**) Image of AMP detection time. (**B**) FL intensity of different detection time at T line.

In general, during the detection process, it takes time to react and diffuse through the NC membrane to reach the T line and C line after loading the sample solution or standard AMP solution. Appropriate detection time can not only save the analysis time but also make sure the reaction is carried out completely. Herein, the reaction time was optimized by using the 50 ng/mL AMP with PBS buffer as the sample in Figure 4. The test strip was placed into the fluorescence and chemiluminescence imaging system and the FL signals from the T line and C line were obtained at various time intervals. The FL intensities of the H-DNA 1 in the T line reached a high at around 3 min and maintained for approximately 20 min. To obtain stability of the data and the completeness of the reaction, five min was selected for balancing operation error and the detection time.

### 3.4. Interference Tolerance of Sample Matrix

To achieve appropriate sensitivity and selectivity, it is essential to evaluate interference tolerance of the developed method. The cross-reactivity and recoveries were applied to evaluate the impact of the sample matrix and external interference. The specificity of the immunoreaction is one of the most important factors in the immunological analysis. In addition to the aptamer, the immunoassay procedure could also affect the selectivity of the reaction. The cross-reactivity of the fabricated system was evaluated in this work. The concentration causing 50% inhibition (IC 50) was used to calculate the cross-reactivity, according to the equation:

$$\mathrm{Cross}-\mathrm{reaction}\%=\frac{[\mathrm{AMP}]\;\mathrm{at}\;\mathrm{IC}\;50}{[M]\;\mathrm{at}\;\mathrm{IC}\;50}$$

where AMP and M refer to the concentration of AMP and the other related antibiotics, respectively. 

Table 1 shows the results of cross-reactivity of the H-DNA 1, T-DNA and C-DNA 1 with some relative antibiotics. The system exhibited 7.40% cross-reactivity with penicillin G (PG), 5.53% with kanamycin (KA), 6.60% with CTC, and <1% with OTC and TC. These results demonstrated that the developed system in this work was sufficiently specific for the AMP detection. 

### 3.5. Selectivity and Sensitivity of the Method

**Figure 5:**
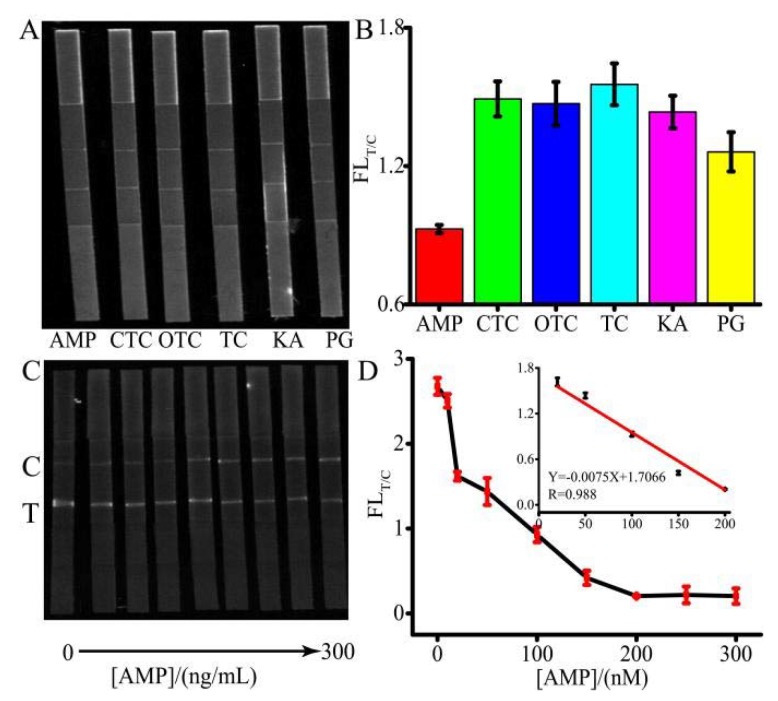
(**A**) Image of the selectivity of the proposal. (**B**) FL_T/C_ indicates the FL intensity ratio of the T-line/C-line. (**C**) Image of the intensity corresponding AMP concentrations. (**D**) The FL intensity ratio was between T-line and C-line with different AMP concentrations. Inset shows a linear range from 10 to 200 ng/L. FL_T/C_ indicates the FL intensity ratio of T-line/C-line.

To examine the selectivity of AMP detection, control experiments were performed using other antibiotics including PG, OTC, KA, TC, and CTC, shown in Figure 5A,B. The concentration of AMP was 20 ng/L, and the concentration of other antibiotics was 200 ng/L. Although there is no standard for the limit of antibiotics in water, according to China’s national standard GBT 22975-2008, the antibiotics residue in milk cannot exceed 32 μg/kg. Therefore, 200 ng/L antibiotics was used. Despite the concentration of the control group being 10-fold higher than that of AMP, the FL_T/C_ ratios of the control group were much higher than that of AMP, which demonstrated that the system had an excellent selectivity. Under the optimized conditions, a quantitative analysis was developed. Figure 5C,D depict that when the concentration of AMP ranged from 0 to 300 ng/L, the linear range changed from 10 to 200 ng/L. According to Figure 5C, the simulated spectrum of the FL related to AMP concentration was shown in Figure S1. A good reproducibility assay of AMP was achieved with RSDs between 2.34% and 8.63% (n = 3). The LOQ was as low as 2.71 ng/L. The sensitivity of the proposed method was comparative or higher than that of the other assays [26,27,28,29,30]. These results implied that the developed method would be a highly sensitive method for AMP detection.

### 3.6. Quantitative Determination of AMP in Hospital Wastewater

Based on the successful development of the CF-LFI method for AMP assay, the quantitative analysis of AMP could be well-achieved. The AMP from ten pre-treated hospital wastewater samples was detected by the developed method, shown in Table 2. No AMP residues were detected in all of the samples. We deduced that little AMP was used, and AMP in hospital wastewater would be adsorbed, hydrolyzed, photolyzed, biodegraded, and so on. Moreover, different concentrations of AMP were spiked into the hospital wastewater according to the standard addition method and determined by the established method. The recoveries were in the range of 80.5% to 105.6% with the RSDs between 3.01% and 10.57% (n = 3), indicating that the proposed method has a great potential for the detection of AMP in the hospital wastewater. Importantly, most recoveries had shown slight change when the sample matrices were treated with five-fold dilution and ten-fold dilution. These results indicated that the sample matrices had minimum interference on the CF-LFI strip. It also finds that sample 5 and sample 8 have greatly changed when they are diluted. The reason is more complex precipitates were found in sample 5 and sample 8 after adding trichloroacetic acid. It further demonstrated that antibiotics could be absorbed or enclosed in matrix. It is concluded that dilution is an effective strategy for sample pretreatment. It also indicated that more effective methods for antibiotics analysis should be developed.

## 4. Conclusions

In summary, a simple method for AMP detection was developed based on the combination of labeled functional nucleic acids and CF-LFI. The designed probes maintained the remarkably specific recognition of AMP and served as a sensor for AMP detection with a good linearity between 10 and 200 ng/L. The LOQ was 2.71 ng/L, which was either more or comparably sensitive than the other developed methods. Moreover, the proposal possessed excellent interference resistance and little external interference. The developed method was successfully applied to detect AMP in hospital wastewater samples and achieved excellent recoveries, showing its high potential for practical applications.

## Figures and Tables

**Table 1:** Cross-reaction of some antibiotics with H-DNA 1, T-DNA and C-DNA 1 in the proposed CF-LFI system.

H-DNA 1, T-DNA and C-DNA 1	Average of Cross- Reactivity (%) (n = 3)	RSD (%) (n = 3)
AMP	100	5.67
PG	7.40	3.56
KA	5.53	3.89
OTC	0.88	2.76
TC	0.67	2.34
CTC	6.60	4.34

**Table 2:** The AMP detection from hospital wastewaters by the developed method.

Sample No.	Detection [AMP] (ng/L)	Spiked [AMP] (ng/L)	Recovery (%)	RSD (%)	Five Dilute Sample with Spiked [AMP] (ng/L)	Recovery (%)	RSD (%)	Ten Dilute Sample with Spiked [AMP] (ng/L)	Recovery (%)	RSD (%)
1	-	20	104.2	9.39	150	104.3	9.67	150	100.5	9.27
2	-	20	94.6	10.57	150	88.7	7.34	150	86.8	6.57
3	-	20	86.1	8.43	150	89.6	7.76	150	92.4	7.12
4	-	20	97.5	7.56	150	87.2	8.43	150	86.7	8.03
5	-	20	82.7	9.81	150	89.7	9.34	150	99.7	8.96
6	-	20	85.9	6.01	150	90.1	9.43	150	87.8	9.65
7	-	20	103.1	5.01	150	103.4	4.52	150	105.6	3.01
8	-	20	88.4	4.67	150	98.7	3.01	150	91.3	3.08
9	-	20	99.4	3.82	150	91.5	7.63	150	92.8	6.37
10	-	20	89.8	4.69	150	90.5	5.64	150	90.5	4.99

## Supplementary Materials

The following are available online at https://www.mdpi.com/2072-666X/11/4/431/s1, Figure S1: The fluorescence spectrum corresponding AMP concentrations was simulated by Image J soft.
