# Supplementary material for: A Fluorescent Sensor-Assisted Paper-Based Competitive Lateral Flow Immunoassay for the Rapid and Sensitive Detection of Ampicillin in Hospital Wastewater
Source: Micromachines (Basel). 2020 Apr 20;11(4):431. doi: 10.3390/mi11040431 (PMC7231327; doi:10.3390/mi11040431)
Supplement: Supplementary file 1 [file micromachines-11-00431-s001.pdf]

Supplementary Materials: A Fluorescent Sensor-assisted Paper-based Competitive Lateral Flow Immunoassay for the Rapid and Sensitive Detection of Ampicillin in Hospital Wastewater

Honggui Lin [1], Feixiang Fang [2] , Jiahui Zang [2], Jianlong Su [2],Qingyuan Tian [1,3], Ranjith Kumar Kankala [2], and Xuexia Lin [2,*]

[1] Fujian Province Key Laboratory of Ship and Ocean Engineering, Marine Engineering College, Jimei University, Xiamen 361021, China; linhongui36@163.com (H.L.); tianqingyuan@jmu.edu.cn (Q,T.)
[2] Department of Chemical Engineering& Pharmaceutical Engineering, College of Chemical Engineering, Huaqiao University, Xiamen 361021, China; zjh19980729@163.com (J.Z.); sujianlong_123@163.com (J.S.); fangfeixiangHQU@163.com (F.F.); ranjithkankala@hqu.edu.cn (R.K.K.)
[3] Marine Engineering College, Dalian Maritime University, Dalian 116026, China
* Correspondence: linxuexia@hqu.edu.cn (X. X. L.)

The illustration of Figure 2

Strip 1：T-DNA,

Strip 2：H-DNA 1 with T-DNA,

Strip 3：H-DNA 1 with C-DNA 1,

Strip 4：H-DNA 1 with AMP,

Strip 5：H-DNA 1 with AMP and C-DNA 1,

Strip 6：H-DNA 1 with AMP and T-DNA,

Strip 7：T-DNA,

Strip 8：H-DNA 2 with T-DNA,

Strip 9：H-DNA 2 with C-DNA 2,

Strip 10：H-DNA 2 with AMP,

Strip 11：H-DNA 2 with AMP and C-DNA 2,

Strip 12：H-DNA 2 with AMP and T-DNA,

Strip 13：C-DNA 1 for 300 ng/L AMP detection,

Strip 14：C-DNA 1 for 200 ng/L AMP detection,

Strip 15：C-DNA 1 for 100 ng/L AMP detection,

Strip 16：C-DNA 1 for 50 ng/L AMP detection,

Strip 17：C-DNA 2 for 300 ng/L AMP detection,

Strip 18：C-DNA 2 for 200 ng/L AMP detection,

Strip 19：C-DNA 2 for 100 ng/L AMP detection,

Strip 20：C-DNA 2 for 50 ng/L AMP detection,

Strip 21：C-DNA 1 with H-DNA 1′ for 150 ng/L AMP detection,

Strip 22：C-DNA 1 with H-DNA 1′ for 200 ng/L AMP detection,

Strip 23：C-DNA 1 with H-DNA 1 for 150 ng/L AMP detection,

Strip 24：C-DNA 1 with H-DNA 1 for 200 ng/L AMP detection.

Table S1. Summary of other methods in recent years to detect AMP.

Assay	Indicator or Method	Linearity Range	Detection Limit	Ref.
HPLC detection	HPLC-UV	1.14~57.2 μM	0.33 μM	6
Fluorescent aptasensor	AuNPs/MBs and nicking enzyme	0.1~100 ng mL^{-1}	0.07 ng mL^{-1}	13
Electrochemical surface plasmon resonance	Electrochemical method and aptamer	2.5~1000 μM	1.0 μM	14
Electrochemical aptamer-based sensor	Alternating current voltammetry	5~5000 μM<br>100~ 5000 μM	1 μM<br>30 μM	27
Electrochemical biosensor	Electrochemical aptasensor（SD-EAS II）	4~150 nM	10 pM	28
Surface Plasmon Resonance	Silver nanoparticles (AgNPs)	25~1200 ng mL^{-1}	10 ng mL^{-1}	29
Piezoelectric sensors	Nanoparticulate molecularly imprinted polymers(NMIPs)	0.1~1.0 μg mL^{-1}	0.09 μg mL^{-1}	30
CE-ECL	CE and ECL	0.050~5.0 μg ml^{-1}	0.018μg mL^{-1}	31

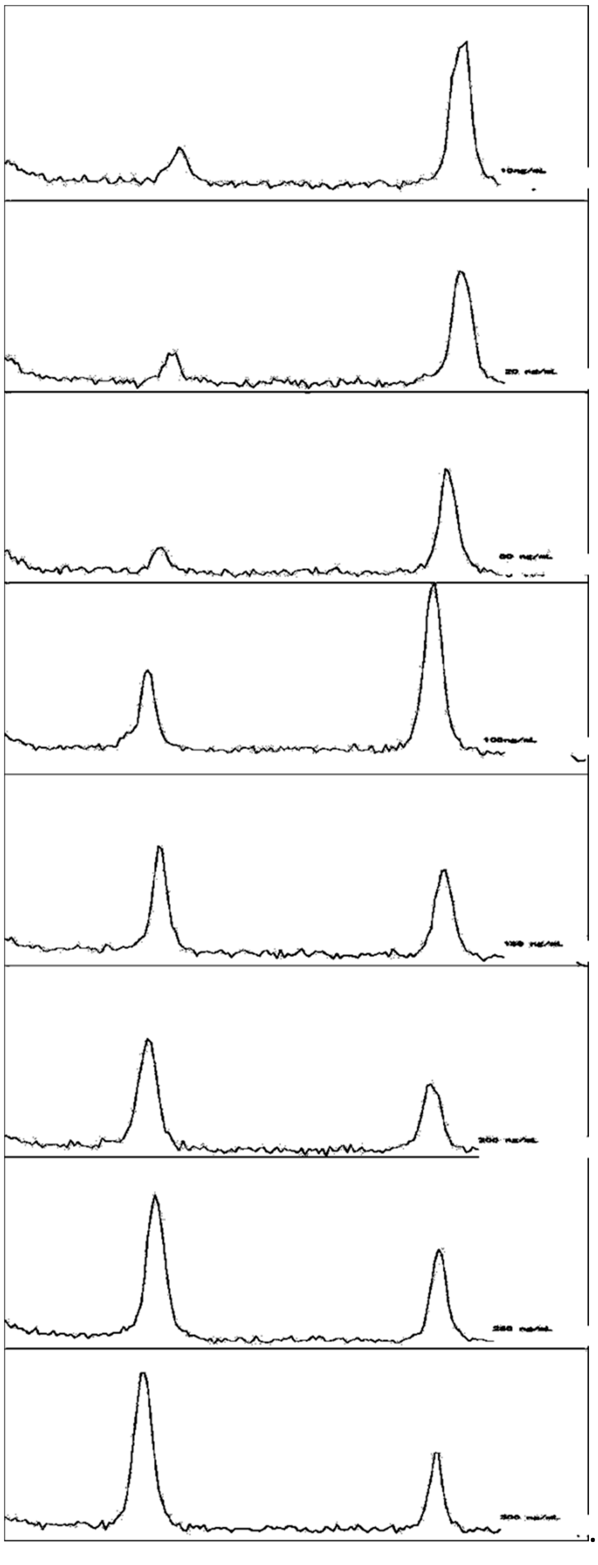

Figure S1. The fluorescence spectrum simulated by Image J soft corresponding AMP concentrations from 10ng/mL to 300 ng/mL. The Concentrations in Figure from top to bottom are 10 ng/mL,20 ng/mL,50 ng/mL,100 ng/mL,150 ng/mL,200 ng/mL,250 ng/mL,300 ng/mL.
